# Simultaneous reconstruction of evolutionary history and epidemiological dynamics from viral sequences with the birth–death SIR model

**DOI:** 10.1098/rsif.2013.1106

**Published:** 2014-05-06

**Authors:** Denise Kühnert, Tanja Stadler, Timothy G. Vaughan, Alexei J. Drummond

**Affiliations:** 1 Department of Computer Science, University of Auckland, Auckland, New Zealand; 2 Department of Biosystems Science and Engineering, ETH Zürich, Basel, Switzerland; 3 Institute of Veterinary, Animal and Biomedical Sciences, Massey University, Palmerston North, New Zealand; 4 Allan Wilson Centre for Molecular Ecology and Evolution, University of Auckland, Auckland, New Zealand

**Keywords:** phylodynamics, Bayesian phylogenetics, birth–death prior, mathematical epidemiology

## Abstract

The evolution of RNA viruses, such as human immunodeficiency virus (HIV), hepatitis C virus and influenza virus, occurs so rapidly that the viruses' genomes contain information on past ecological dynamics. Hence, we develop a phylodynamic method that enables the joint estimation of epidemiological parameters and phylogenetic history. Based on a compartmental susceptible–infected–removed (SIR) model, this method provides separate information on incidence and prevalence of infections. Detailed information on the interaction of host population dynamics and evolutionary history can inform decisions on how to contain or entirely avoid disease outbreaks. We apply our birth–death SIR method to two viral datasets. First, five HIV type 1 clusters sampled in the UK between 1999 and 2003 are analysed. The estimated basic reproduction ratios range from 1.9 to 3.2 among the clusters. All clusters show a decline in the growth rate of the local epidemic in the middle or end of the 1990s. The analysis of a hepatitis C virus genotype 2c dataset shows that the local epidemic in the Córdoban city Cruz del Eje originated around 1906 (median), coinciding with an immigration wave from Europe to central Argentina that dates from 1880 to 1920. The estimated time of epidemic peak is around 1970.

## 1. Introduction

The fast evolution of RNA viruses poses a challenge: their evolutionary processes are subjected to ecological dynamics that occur on the same timescale [1,2]. Therefore, a credible model of virus evolution has to take time-dependent ecological processes into account. In this work, we present a method for Bayesian inference under a phylodynamic model that simultaneously estimates epidemiological parameters and reconstructs phylogenetic history.

Recent developments have provided us with extensive amounts of genomic data. In the case of human immunodeficiency virus (HIV), a number of countries, such as Switzerland [3] and the UK [4], have sampled a large fraction of HIV-infected residents. Analysis of such datasets requires careful validation of methods. For example, standard coalescent models require the population size to be constant or to vary deterministically. To accommodate stochastic population size changes within phylogenetic reconstruction, a tree prior based on the birth–death process [5,6] has been developed by [7].

An extension of Stadler's birth–death–sampling model, the birth–death skyline plot (BDSKY) [8] allows for serially sampled data and rate changes over time. These rate changes through time may reflect environmental changes, for example, new treatment strategies or behaviour changes at different points in time.

Host population dynamics can strongly affect viral transmission and evolution [1]. Therefore, modelling the underlying host population through compartmental models not only provides additional information on the viral outbreak, but also informs the estimates for evolutionary reconstruction. We show here that the BDSKY plot can be parametrized to enable the underlying population dynamics to be modelled as a compartmental susceptible–infected–removed (SIR) model, a classic epidemiological model, which accounts for changing host population composition [9].

In the birth–death SIR (BDSIR) model presented in this paper, we assume that a gene genealogy, i.e. the phylogeny connecting the sampled sequences, represents the past transmission history of the hosts (note that of course this transmission history is incomplete as many infected hosts may not be sampled). That is, an infected host corresponds to a portion of a single lineage in the phylogeny, and of the two child branches produced at a branching node, one represents the continuation of the donor infection, whereas the other represents the new recipient.

We introduce the BDSIR model for estimating epidemiological parameters, for example the basic reproductive number based on sequence data. The model approximates a classic stochastic SIR model. In summary, our method works as follows. Trajectories of the number of susceptible, infected and removed individuals are provided by the SIR model. Based on the trajectory of infected individuals, the average transmission rate in short time intervals throughout the epidemic is determined. The likelihood of the proposed sampled tree connecting the sequence data is then obtained based on these piecewise constant transmission rates using the birth–death skyline model. This BDSIR model is implemented into the Bayesian software framework BEAST2 (http://beast2.cs.auckland.ac.nz).

We then perform a simulation study showing the accuracy of the BDSIR model. Applied to HIV-1 type B sequences sampled in the UK, the method gives insight into the epidemic features of five local epidemics. Although it is common to model the infection dynamics of HIV with non-recovery (SI) models, here we model it as an SIR model. In countries like the UK, behaviour changes and commencement of treatment are expected to coincide with the sampling of HIV-positive individuals, which can imply the removal of the individual from the infectious pool [10]. Finally, we apply the method to a set of hepatitis C virus (HCV) type 2c sequences from the city of Cruz del Eje (CdE) in the Argentinian province Córdoba. European immigration likely caused the outbreak of this local epidemic. Many of the immigrants came from Italy, where HCV subtype 2c is also common [11]. The epidemic appears to have peaked around 1970 and to be in its decline now.

## 2. Material and methods

### 2.1. Stochastic epidemiological models

Infectious disease epidemics are classically modelled through compartmentalization into a number of host compartments, such as susceptible, infected and removed individuals (SIR model), where a susceptible individual moves to the infected compartment upon infection, and an infected individual moves to the removed compartment upon removal/recovery. Such a model may be extended by assuming an exposed class (SEIR model), altered by assuming no removal/recovery (SI model) or no immunity of recovered individuals (SIS model) [12].

In the following, we formalize a stochastic epidemiological SIR model, which we will use for phylogenetic inference assuming an unstructured population. It is relatively straightforward for other unstructured compartmental epidemiological models to be placed into a stochastic framework for phylogenetic analysis in the same way.

In terms of its reaction kinetics, a stochastic SIR model has the following scheme:2.1
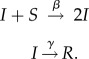


An individual in the infected compartment *I* infects a susceptible individual *S* at a mass-action infection rate of *β*. An infected individual *I* recovers at recovery rate *γ*.

Typically, such SIR models are formalized through a system of ordinary differential equations, which represent a mean field approximation of the expected number of susceptible, infected and removed individuals through time, of a stochastic model, with *n*_S_(0) susceptible individuals, *n*_I_(0) = 1 infected and *n*_R_(0) = 0 removed individuals as initial conditions at time 0:
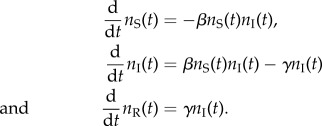


Stochasticity plays a significant role in viral epidemics, especially at the very beginning of an epidemic. Although large epidemics can be described by deterministic models once they are established, these deterministic models must condition on the time at which the exponential growth phase of the epidemic begins, as this starting time impacts the timing of every event thereafter.

Hence, we employ stochastic epidemiological models here. Under the stochastic SIR model, an infected individual infects a susceptible individual with rate *β* and recovers with rate *γ*.

In most epidemics, we only observe a proportion *s* of the recoveries. We can include this by adding another reaction to equation (2.1)2.2
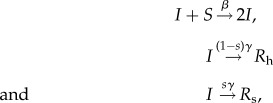
where we distinguish between *hidden* or unobserved recoveries *R*_h_ and *sampled* or observed recoveries *R*_s_. The *sampling proportion s* with 0 ≤ *s* ≤ 1 is the probability of a recovery being observed, and thus the expected proportion of recoveries observed. This infection process, where only some recoveries are observed, is the basis for connecting nonlinear epidemiological models to phylogenetic data.

Molecular sequence data from infected hosts, which are used to infer the phylogenetic tree, are often sampled sequentially through time. In our model, we account directly for this sequential sampling as an infected individual is sampled with rate *ψ* = *sγ*, and upon sampling the individual moves to the removed class (owing to e.g. successful treatment or behaviour change).

**Figure 1.**
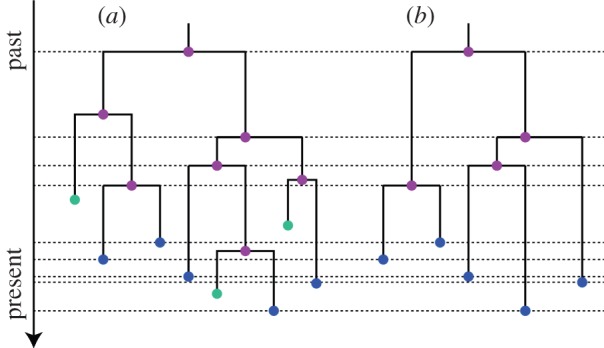
Sequentially sampled birth–death–sampling tree. (*a*) Full transmission tree with birth (internal nodes, purple), sampling (leaves meeting dotted lines, blue) and death (remaining leaves, green) events. (*b*) Full tree pruned to include only observed, i.e. sampled individuals. (Online version in colour.)

The stochastic SIR model with transmission rate *β*, recovery rate *γ*, sampling proportion *s*, population size *n*_S_(0) and timespan of the epidemic being *T* induces a distribution of full transmission chains through time (i.e. who infected whom). The sampled tree (or sampled transmission chain) results from the full transmission chain by pruning all non-sampled lineages, i.e. the tips of the sampled tree are the sampled individuals (figure 1). The trajectories of the SIR model are the time series of the number of susceptible, infected and removed individuals through time.

Note that we assume the host population size *N* = *n*_S_(*i*) + *n*_I_(*i*) + *n*_R_(*i*) to be constant over time, in which case our population-dependent model (transmission term *βn*_S_*n*_I_) is equivalent to a frequency-dependent model (transmission term (*β/N*)*n*_S_*n*_I_).

### 2.2. Incorporating stochastic epidemiological models into phylogenetics

**Figure 2.**
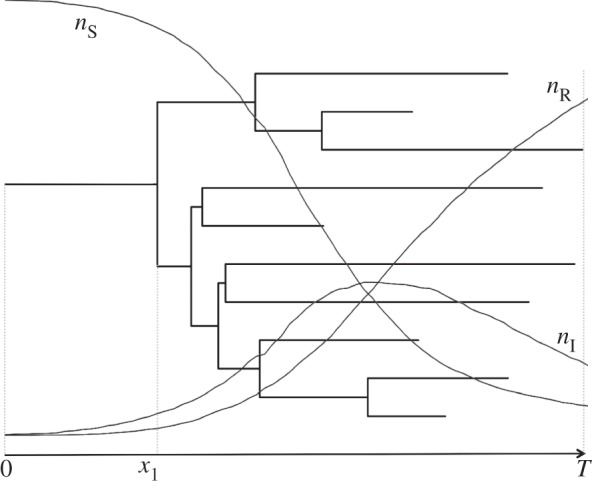
An epidemic starts at time 0, giving rise to the genealogy rooted at time *x*_1_, and trajectories for the number of susceptible (*n*_S_), infected (*n*_I_) and removed (*n*_R_) individuals. The last sampled tip determines the end of the observed epidemic at time *T*.

We do not have information about unobserved individuals, i.e. we cannot expect to infer the full transmission chain. However, based on sequenced data *D* from a sample of infected individuals, we aim at inferring the sampled transmission tree 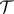, the evolutionary parameters *θ*, the SIR trajectories
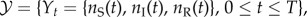
(where 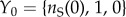, i.e. initially all individuals are susceptible, apart from one individual, which is infected) and the epidemiological parameters 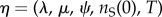, where *λ* = *βn*_S_(0), *μ* = (1−*s*)*γ* and *ψ* = *sγ*, in a Bayesian framework (figure 2). In particular, we want to infer the posterior distribution of trees, trajectories and parameters,
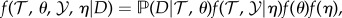
with 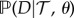 being the likelihood of the sequences given a tree (which can be calculated efficiently with Felsenstein's pruning algorithm [13]) and *f*(*θ*), *f*(*η*) being the prior distributions on the parameters. Furthermore, the inference requires the expression for the joint probability of the sampled tree and the trajectories given the epidemiological parameters, 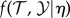. We rewrite
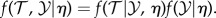

The right-hand side of the equation is the probability density of a sampled transmission tree given the trajectories and epidemiological parameters, multiplied by the probability density of the trajectories given the epidemiological parameters. Both terms must be determined so that we can do Bayesian phylogenetic inference under the stochastic SIR model.

Instead of calculating 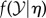, we can simulate a trajectory given the epidemiological parameters *η* in each Markov chain Monte Carlo (MCMC) step (for details see electronic supplementary material, text S2). Given the simulated trajectory, it remains to calculate 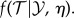


For calculating the probability density of a sampled tree, we note that when conditioning on the full trajectories, we have 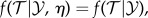 and the probability of a sampled tree given the trajectories, 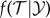, is a product where at each event in the trajectories we multiply by the probability of the event having happened in the sampled tree if it coincided with a tree event, and multiply by the probability of the event having not happened in the sampled tree if it did not coincide with a tree event. Thus, theoretically we can both simulate trajectories and evaluate the tree probability 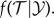 For large population sizes (i.e. large *n*_S_(0)), the number of events will grow very large, thus both trajectory simulations and tree likelihood calculation will become very slow. Therefore, we do not substitute 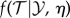 by 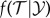. Instead, we approximate both the simulation and likelihood calculation by discretizing time. With the simulation techniques described in the electronic supplementary material, text S2 we simulate at discrete time points *t*_1_, *t*_2_, *…* , *t_m_* where 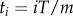, the number of susceptible, infected and removed individuals, i.e. we have trajectories 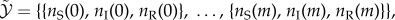 with the initial value at time 0 being {*n*_S_(0), 1, 0}. Then, we need to calculate 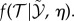


We note that so far, for *m* → *∞*, convergence to the exact probability densities holds. However, we did not find an efficient way to calculate the required probability density 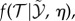 thus we introduce an approximation below, yielding the BDSIR model, which does not converge to the exact probability density, but turns out to be efficient and accurate.

We sample trajectories 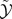 from 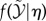 with a *τ*-leaping algorithm (see the electronic supplementary material, text S2).

### 2.3. The birth–death SIR model

The BDSIR model is an approximate stochastic epidemiological model in phylogenetics. We approximate the stochastic SIR model by the BDSIR model, leading to an efficient way to calculate approximately the likelihood of the phylogeny given the epidemiological time series and parameters 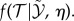


In the BDSIR model, the epidemiological trajectories are defined stochastically by the SIR model with constant population size (*n*_S_(*i*) + *n*_I_(*i*) + *n*_R_(*i*)) and simulated using the *τ*-leaping approach described in the electronic supplementary material, text S2. Simulations are started with an initial number of susceptibles, *n*_S_(0), and last for time *T*. At equally spaced time points *t*_1_, *…* , *t_m_*, the values of the trajectories *n*_S_(*i*), *n*_I_(*i*), *n*_R_(*i*) are recorded, yielding 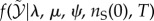. The trajectories converge to SIR trajectories 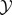 for *m* → *∞*.

Under the BDSIR model, a sampled tree is induced by a so-called BDSKY plot [8] given the discrete time trajectories 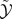 as follows. The transmission rate *λ*_*i*_ during time interval [*t_i_*, *t_i_*_+__1_) is parametrized by *λ*_*i*_ = *βn*_S_(*i*), where *β* is the epidemiological transmission rate and *n*_S_(*i*) is the number of susceptibles at time *t_i_*. The recovery rate *γ* and sampling fraction *s* are constant through time. Piecewise constant transmission rates in the BDSIR model allow the calculation of the likelihood of a sampled tree 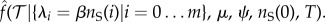 This likelihood is given by the probability density of the BDSKY plot (for a derivation of the probability density see ([8], Theorem 1)) with piecewise constant transmission rate *λ*_*i*_ = *βn*_S_(*i*) and constant death and sampling rate *μ* and *ψ*, respectively. The equation for the probability density of a sampled tree is stated in the electronic supplementary material, text S1.

In the BDSIR model, we approximate the calculation of the posterior distribution under the stochastic SIR model,2.3
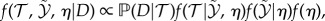
by using2.4
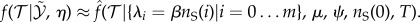
While 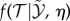 converges to 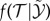 as *m* → *∞*, the approximation (equation (2.4), right-hand side) does not: under the skyline plot, we only specify the transmission rates based on 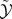. Based on these time-varying transmission rates, we calculate the likelihood of the tree by integrating over all possible trajectories 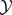 yielding the given tree (instead of conditioning on 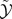).

### 2.4. Markov chain Monte Carlo implementation of the birth–death SIR model

We implemented equations (2.3) and (2.4) into BEAST for joint phylogenetic tree and epidemiological parameter inference (code and examples can be downloaded from http://code.google.com/p/phylodynamics). The prior distribution 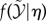 in equation (2.3) is subsumed in the proposal kernel of an MCMC implementation, so that a new trajectory 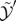 is proposed by simulation, whenever a new *η’* is proposed giving a joint proposal kernel of
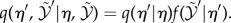


Therefore, BDSIR uses an independence Metropolis–Hastings (MH) sampler, as introduced by Stephens *et al*. [14] and subsequently studied by many others, e.g. [15,16]. This leads to the Metropolis–Hastings acceptance ratio [17]
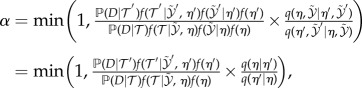
where *η′* denotes the new proposal of parameters *η*, etc. The factor 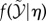 is implicitly included in the posterior through independence sampling of the time series 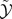. The proposal is rejected if at any of the times *t_i_*, *i* = 0 *…* *m*, the number of infected individuals in the proposed trajectory is less than the corresponding number of lineages in the phylogenetic tree.

In our simulation study, we show that the approximation for the tree likelihood (equation (2.4)) is suitable, by illustrating that we can infer parameters from simulated phylogenies with high accuracy. Thus, by applying our BDSIR model to virus sequence data from different infected individuals throughout an epidemic, the phylogenetic tree can be estimated jointly with the epidemiological parameters *η*. The choice of Bayesian parameter prior distributions is facilitated by the parametrization of the epidemiological parameters as the basic reproduction ratio 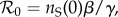 the rate at which infected individuals become non-infectious *γ*, the sampling proportion *s*, the initial susceptible population size *n*_S_(0) and the length of the epidemic *T*.

### 2.5. Simulation study

Using simulations, we explore how well the BDSIR model performs when inferring parameters based on simulated trees. In Stage 1, we simulate 100 SIR trees based on the reaction scheme (2.2) with *n*_S_(0) = 999, *β* = 0.00075, *γ* = 0.30 and *s* = 1/6 (i.e. 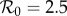). Each simulated tree has 100 tips. Then, we set up an analysis to re-estimate the simulation parameters for each of the simulated trees. In this second stage, the tree and the duration *T* of the epidemic are fixed; they represent the data from which we estimate the epidemiological parameters.

Stage 2 comprises two × two sets of analyses: in the first two sets, we fixed the sampling proportion *s* as we showed in [8] that *λ*, *γ* and *s* correlate; in the second two sets, we estimated *s*. In each set of two, the initial number of susceptible individuals *n*_S_(0) is firstly fixed to the true value and secondly all parameters including *n*_S_(0) are estimated. We chose *m* = 100 equidistant time points *t*_1_, *t*_2_, *…* , *t_m_* to discretize the epidemic trajectories. For comparison, we also estimate the rates of the second two sets (i.e. estimating *s*) with (i) the BDSKY model [8] with piecewise constant effective reproduction ratio and (ii) the birth–death–sampling model with constant effective reproduction ratio [18].

While the birth–death–sampling model characterizes the tree-generating process through constant birth, death and sampling rates, these rates can change in a piecewise fashion in the BDSKY model. Both methods differ from the BDSIR model in that they do not explicitly parametrize the underlying host population dynamics. We compare the estimated parameters to the true parameter values. In particular, we focus on the *basic reproduction ratio* 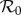 (the average number of secondary infections in a completely susceptible population) and the *effective reproduction ratio* (the average number of secondary infections in the current population).

The BDSIR method estimates the basic reproduction ratio as 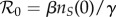. BDSKY estimates the effective reproduction ratio 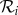 for each time interval 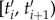. We chose 10 intervals for the BDSKY analysis such that 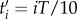. We obtained the ‘true’ effective reproduction ratio from the Stage 1 simulations of the SIR trees (as well as the estimates for BDSIR) by computing the averaged effective reproduction ratios 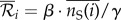, *i* = 1..10, (where 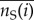 is the mean number of susceptible individuals, given by true trajectory 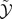 in time interval 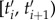).

Relative error, bias and highest posterior density (HPD) width served as measures of precision and accuracy. We define the relative error as
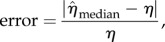
the relative bias as
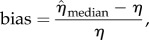
and finally the 95% relative HPD width is defined as
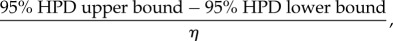
where *η* is the true parameter and 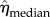 is the posterior median value of the parameter.

The Bayesian prior distributions used in Stage 2 are given in table 1.

**Table 1.** Prior distributions for the re-estimation of SIR parameters from simulated trees (equal priors applied in BDSIR and birth–death–sampling analyses) and for data analyses.

analysis	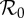	*γ*	*s*	*n_S_*(0)	*T*	*ρ*
simulated SIR	Log*N*(1,1)	Log*N*(−0.5,1)	Beta(2,10)	Log*N*(7,1)	—	—
HIV data UK	Log*N*(0.5,0.5)	Log*N*(−1,0.75)	Beta(1,1)	Log*N*(7,1.25)	Unif(0,1000)	—
HCV data CdE	Log*N*(0,2)	Log*N*(−0.5,1.25)	—	Unif(0,30000)	Unif(0,1000)	Unif(0,1)

### 2.6. HIV-1 type B in the UK

A set of molecular sequences sampled from HIV-1 type B infected individuals in the UK have been grouped into five phylogenetic clusters [19]. Sampled between 1999 and 2003, these clusters represent a suitable example dataset for the analysis under the BDSIR model. The clusters comprise 41, 62, 29, 26 and 35 sequences, respectively, and correspond to clusters 1–4 and 6 in the original analysis. Each cluster is considered as a sample from a local sub-epidemic. Our model explicitly accounts for the incomplete sampling of the local epidemics. These clusters have been identified based on a phylogenetic neighbour-joining tree that was constructed from 3429 HIV-1 subtype B pol gene sequences from the UK and throughout the world. Note that the clusters are therefore not randomly sampled, and we also cannot guarantee that the sample sets are truly isolated transmission clusters. Although this identification of transmission clusters is common practice, we point out that it may introduce a bias.

Note that we use an SIR model, although true recovery in the literal sense does not (yet) occur in HIV-infected individuals. This is reasonable in countries like the UK, owing to changes in behaviour as well as the effects of combination drug therapy, which can reduce viral load to undetectable levels, severely diminishing the risk of further transmissions and, hence, implying removal of the individual from the infectious pool. However, during the earlier part of the study period, i.e. before the introduction of HAART, this does not hold. Furthermore, modelling the HIV host population dynamics as a closed SIR compartmental model requires assuming that the times at which individuals move between compartments are exponentially distributed and that the host population size remains constant over time. Another implicit simplifying assumption is that infected individuals are constantly infectious.

The phylodynamic analysis employed a general time reversible substitution model with gamma distributed rate heterogeneity and a proportion of invariant sites (GTR + *Γ* + I), and all parameters were estimated jointly apart from the substitution rate, which was fixed to 2.55 × 10*^−^*^3^, as in [19]. Before 1999, we assume the sampling proportion *s* to be zero, as all samples were collected between 1999 and 2003.

### 2.7. HCV type 2c in Argentina

We analyse a set of 44 HCV type 2c sequences (NS5B region) that were sampled in 2004 during a survey in the city of CdE, in Córdoba province, Argentina. According to the survey, the 44 sequences included here represent roughly 2.8% of the HCV-2c infected individuals in CdE, which has a population size of about 35 000 and a proportion of 90% genotype 2c infections out of all HCV-positive patients encountered during the survey [20]. Genotype 2c was probably introduced to Argentina during a European immigration wave between 1880 and 1920 [11]. A superset of these data (with additional samples from Córdoba province) were recently analysed by Dearlove & Wilson [21], and in their model comparison they found that the SIR model is most suitable for these data. The analysis employed a GTR + *Γ* + I substitution model and a strict clock model with the substitution rate fixed to 0.58 × 10*^−^*^3^ [22]. As all sequences were sampled at one time point (i.e. homochronously), we model the sampling process through a sampling probability *ρ* [8]. This means that at the end of the tree (e.g. in 2004) each infected individual was sampled with probability *ρ*.

In all analyses, SIR trajectories were sampled at *m* = 100 intervals. Table 1 gives the choice of Bayesian prior distributions for the analyses.

## 3. Results

### 3.1. Simulation study

**Figure 3.**
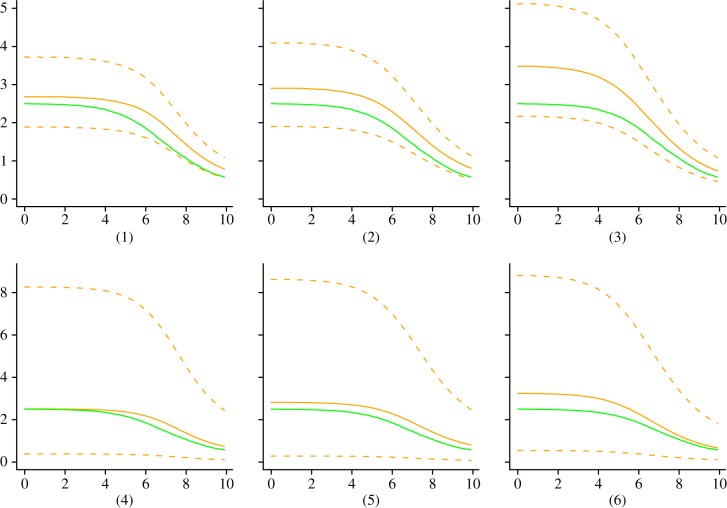
Reconstructed effective reproduction ratio from simulated SIR trees. True trajectory (green/dark) versus estimated trajectory (orange/light) with 95% HPD (dashed lines). Random sample of the 100 reconstruction results shown with *n*_S_(0) fixed to the true value (1–3) and estimated (4–6). Estimation of *n*_S_(0) throughout the phylodynamic reconstruction results in broader HPD intervals. (Online version in colour.)

We investigated the accuracy of our method through a simulation study. Based on reaction scheme (2.2), 100 serially sampled trees were simulated and then used for re-estimation of the simulation parameters. All four sets of analyses, (1) BDSIR with fixed *n*_S_(0), (2) BDSIR, (3) BDSKY with *m* = 10 intervals (i.e. 9 rate changes) and (4) birth–death–sampling, resulted in accurate estimates of the corresponding simulation parameters or their time-averages (tables 2–7). Figure 3 shows trajectories of the reconstructed reproduction ratio for three simulations (randomly chosen from the set of 100 simulations). As one would expect, estimating the initial number of susceptible individuals *n*_S_(0) rather than fixing it to the true value results in broader 95% HPD intervals.

**Table 2.** BDSIR simulation results (*n*_S_(0) fixed). Posterior parameter estimates and accuracy obtained from 100 simulated trees with 100 tips sampled sequentially through time. *n*_S_(0) is fixed to the true simulation value. For each parameter, the median over the 100 medians/errors/biases/HPD widths/HPD accuracies is provided.

	truth	median	error	bias	relative HPD width	95% HPD accuracy (%)
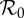	2.50	2.74	0.13	0.10	0.81	100.00
*γ*	0.30	0.26	0.16	–0.14	0.90	99.00
*s*	0.17	0.22	0.34	0.32	1.90	100.00

**Table 3.** BDSIR simulation results (*n*_S_(0) fixed). Computed averages for the effective reproduction number from 100 simulated trees with 100 tips sampled sequentially through time. *n*_S_(0) is fixed to the true simulation value. For each parameter, the median over the 100 medians/errors/biases/HPD widths/HPD accuracies is provided. The averages 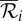 for *i* = 1…10 were computed from the estimated trajectories, 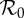, *γ* and *s*.

	truth	median	error	bias	relative HPD width	95% HPD accuracy (%)
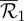	2.49	2.76	0.15	0.12	0.81	100.00
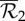	2.48	2.73	0.15	0.12	0.81	100.00
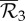	2.45	2.69	0.16	0.13	0.80	100.00
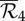	2.39	2.58	0.18	0.16	0.80	99.00
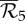	2.25	2.42	0.24	0.24	0.79	98.70
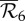	2.00	2.12	0.39	0.39	0.77	97.20
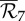	1.63	1.72	0.72	0.72	0.77	94.70
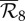	1.23	1.32	1.27	1.27	0.77	87.50
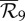	0.89	0.98	2.17	2.17	0.81	83.90
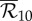	0.65	0.76	3.33	3.33	0.86	80.67

**Table 4.** BDSIR simulation results (*n*_S_(0) estimated). Posterior parameter estimates and accuracy obtained from 100 simulated trees with 100 tips sampled sequentially through time. *n*_S_(0)is estimated in each analysis. For each parameter, the median over the 100 medians/errors/biases/HPD widths/HPD accuracies is provided.

	truth	median	error	bias	relative HPD width	95% HPD accuracy (%)
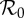	2.50	2.63	0.12	0.05	0.87	100.00
*γ*	0.30	0.29	0.13	−0.05	1.21	100.00
*s*	0.17	0.18	0.19	0.11	1.95	100.00
*n*_S_(0)	999.00	1900.68	0.90	0.90	5.44	100.00

**Table 5.** BDSIR simulation results (*n*_S_(0) estimated). Computed averages for the effective reproduction number from 100 simulated trees with 100 tips sampled sequentially through time. *n*_S_(0) is estimated in each analysis. For each parameter, the median over the 100 medians/errors/biases/HPD widths/HPD accuracies is provided. The averages 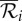 for *i* = 1…10 were computed from the estimated trajectories, 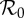, *γ* and *s*.

	truth	median	error	bias	relative HPD width	95% HPD accuracy (%)
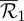	2.49	2.64	0.13	0.07	3.10	100.00
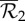	2.48	2.62	0.13	0.08	3.10	100.00
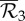	2.45	2.58	0.14	0.09	3.10	100.00
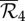	2.39	2.47	0.15	0.12	3.08	100.00
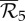	2.25	2.33	0.20	0.19	3.07	100.00
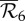	2.00	2.07	0.34	0.34	3.04	100.00
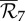	1.63	1.70	0.65	0.65	3.06	100.00
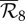	1.23	1.31	1.19	1.19	3.11	100.00
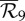	0.89	0.97	2.05	2.05	3.26	100.00
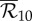	0.65	0.75	3.16	3.16	3.47	100.00

**Table 6.** Birth–death skyline simulation results. Birth–death skyline posterior parameter estimates and accuracy obtained from 100 simulated trees with 100 tips sampled sequentially through time. Rate changes are allowed among 10 equidistant intervals. For each parameter, the median over the 100 medians/errors/biases/HPD widths/HPD accuracies is provided.

	truth	median	error	bias	relative HPD width	95% HPD accuracy (%)
*γ*	0.30	0.23	0.24	–0.23	0.28	99
*s*	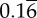	0.24	0.46	0.44	0.40	100
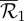	2.49	2.49	0.33	–0.003	5.81	100
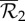	2.48	2.53	0.32	0.02	4.88	99
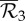	2.45	2.72	0.30	0.11	4.13	99
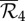	2.39	2.73	0.27	0.14	3.33	98
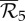	2.25	2.65	0.24	0.17	2.77	97
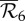	2.00	2.31	0.23	0.14	2.24	95
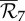	1.63	1.85	0.27	0.11	1.90	92
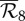	1.23	1.42	0.32	0.12	1.69	91
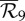	0.89	1.01	0.32	0.11	1.58	98
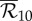	0.65	1.16	0.77	0.77	2.26	97

**Table 7.** Birth–death–sampling simulation results. Birth–death–sampling posterior parameter estimates and accuracy obtained from 100 simulated trees with 100 tips sampled sequentially through time. Rates are assumed constant over time. For each parameter, the median over the 100 medians/errors/biases/HPD widths/HPD accuracies is provided.

	truth	median	error	bias	relative HPD width	95% HPD accuracy (%)
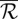	1.86	1.63	0.13	–0.12	1.17	92
*γ*	0.30	0.30	0.08	–0.002	0.52	100
*s*	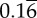	0.17	0.04	0.02	0.38	100

The epidemic dynamics were recovered well for all three analysis sets (1)−(3). A slight positive bias in the estimates of the reproduction ratios is observed, which we speculate is owing to the approximation employed by this method. This bias is small for low reproduction ratios (*R*_0_ < 5), where demographic stochastic effects are relevant, and the coverage properties of the estimator show that the uncertainty in the estimates is accurate. This bias increases with higher *R*_0_ (data not shown), suggesting that the BDSIR method is the most appropriate one for modelling epidemics with low to moderate reproduction ratios (*R*_0_ < 10). The effective reproduction ratio 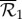 near the origin of the epidemic is estimated with the smallest bias among all 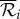, *i* = 1*…*10, respectively. Analysis under BDSKY results in the broadest relative HPD for 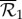. Moving towards the present, the HPD interval widths for BDSKY mainly decrease. The uncertainty in the epidemic dynamics suppresses this effect in the BDSIR analyses: the relative HPD widths of the computed averages 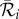 vary only slightly among the time intervals. Overall, the BDSIR analyses with *n*_S_(0) fixed to the true value obtains the narrowest HPD intervals, yet error rates and HPD accuracy are best when *n*_S_(0) is estimated.

The birth–death–sampling model, which is equivalent to a one-dimensional BDSKY model, estimates the time averaged reproduction ratio accurately with quite narrow HPD intervals, suggesting it may be a reasonable method for inference in scenarios where the epidemic dynamics over time are not important.

As shown by [8], the parameters 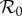, *γ* and *s* of a birth–death–sampling tree prior are correlated. Therefore, we performed an additional set of simulations in which the sampling proportion *s* is fixed to the true value. As expected, this results in narrower HPD intervals with accurate estimates of 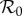 and *γ*. The HPD for the initial number of susceptible individuals *n*_S_(0) contains the true value, but is fairly wide as before (electronic supplementary material, tables S1–S4). These simulation results suggest that additional information about the pathogen under investigation can improve the parameter estimates of the BDSIR analysis. In the case of HIV, for example, many countries have good estimates of how much of the infected population has been sampled.

### 3.2. HIV-1 type B in the UK

We apply the BDSIR method to five HIV-1 clusters sampled between 1999 and 2003, mainly (85%) from men having sex with men around London [19]. Bayesian estimates for the epidemiological parameters and time to the most recent common ancestors of the clusters are summarized in table 8.

**Table 8.** HIV-1 type B from the UK: Bayesian parameter estimates. Bayesian parameter estimates and HPD intervals (in parentheses) from phylodynamic analysis of five HIV-1 type B cluster from the UK.

cluster	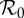	*γ*	*s*	*n*_S_(0)	root of the tree (year)	origin of the epidemic (year)
1	3.22 (2.18–4.27)	0.30 (0.15–0.47)	0.68 (0.25–1)	880 (142–3592)	1986 (1983–1988)	1983(1978–1987)
2	2.45 (1.53–3.68)	0.17 (0.06–0.35)	0.47 (0.1–0.96)	1745 (190–8892)	1983 (1979–1986)	1978 (1968–1984)
3	1.90 (1.22–2.78)	0.20 (0.09–0.39)	0.68 (0.27–1)	1540 (153–8558)	1985 (1981–1988)	1978 (1962–1986)
4	2.62 (1.45–4.29)	0.15 (0.06–0.31)	0.38 (0.06–0.93)	1921 (128–11007)	1987 (1983–1990)	1981 (1970–1988)
5	3.17 (1.73–5.43)	0.15 (0.06–0.31)	0.21 (0.02–0.79)	2862 (183–16 909)	1986 (1981–1989)	1983 (1975–1989)

**Figure 4.**
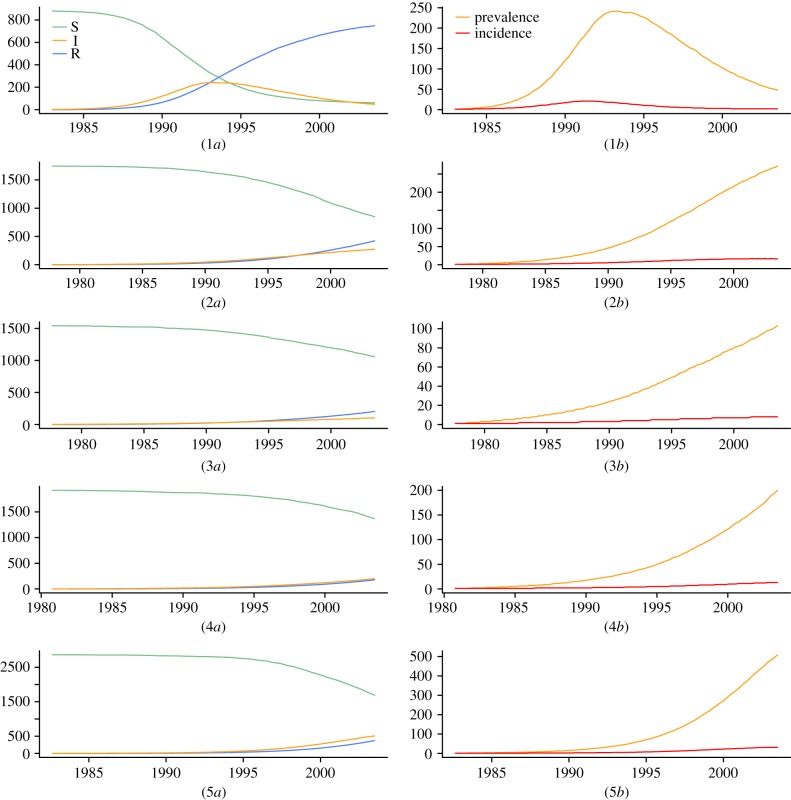
SIR trajectories and incidence of HIV-1 clusters from the UK. Bayesian posterior mean trajectories for clusters (1–5): the overall SIR dynamics (*a*) show at what stage in the epidemic each cluster was sampled. Zooming into the number of infecteds, i.e. the prevalence over time in (*b*) enables comparison to the incidence. (Online version in colour.)

**Figure 5.**
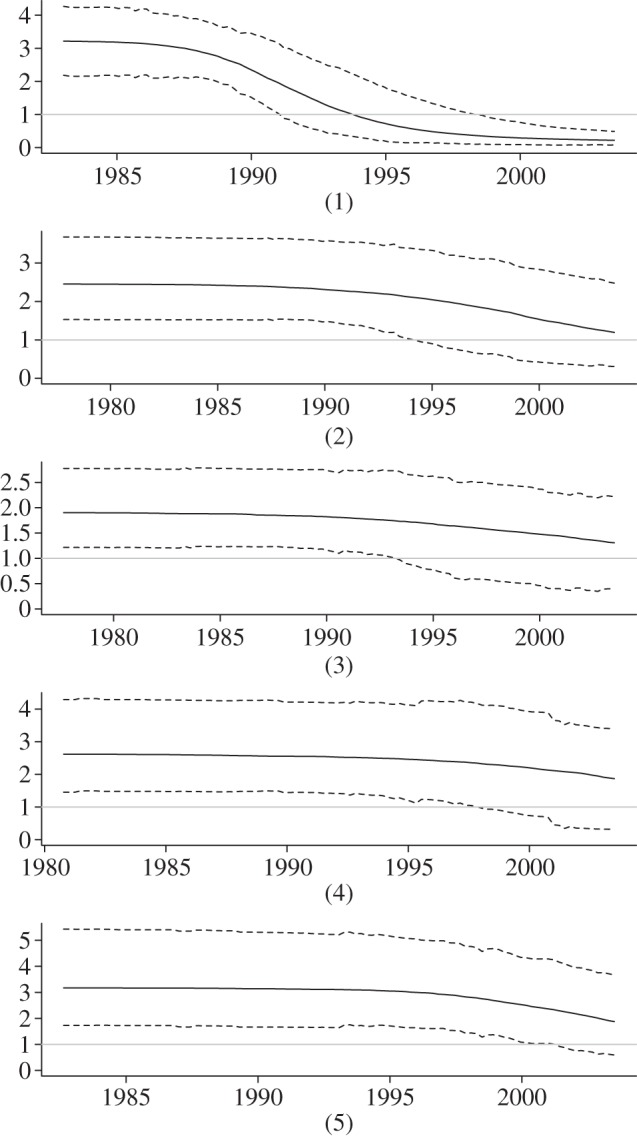
HIV from the UK: reconstructed effective reproduction ratio over time. Median effective reproduction ratio for each cluster, computed from the posterior birth–death rates and SIR trajectories. Dotted lines show the 95% HPD interval.

Our results suggest that the local epidemics corresponding to each of the five genetic clusters have been sampled at varying epidemic stages. Figure 4 shows the posterior medians of the epidemic time series and suggests that cluster 1 is the only cluster that has gone through the largest part of its local epidemic. A single sampled trajectory for each cluster demonstrates the stochastic noise in the epidemics (electronic supplementary material, figure S1). At the end of the sampled interval, the pool of susceptible individuals of this cluster has been depleted nearly completely. On the other hand, the other four clusters are just before or at the peak of the local epidemic. The estimated depletion of susceptible individuals especially in cluster 2 indicates that those epidemics have progressed fairly far and one would expect a decline in the number of infected individuals soon after the end of the sampled interval. These dynamics can also be seen in the plots of the average effective reproduction ratio 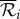 over time (figure 5).

The basic reproduction ratio 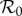 estimated from these clusters ranges from 1.90 (95% HPD: 1.22–2.78) in cluster 3 to 3.22 (95% HPD 2.18–4.27) in cluster 1. There are significant differences in the estimated 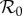 values across the five clusters, despite them all sharing the same prior, which demonstrates that the sequence data contain substantial information about the basic reproduction ratio. These results are robust to a change of the 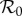 prior distribution (data not shown). Median estimates of the rate to become non-infectious range from 0.15 to 0.30, indicating an average infectious period of about 3−7 years in these clusters.

In all clusters the estimates of the sampling proportion *s* and the initial number of susceptible individuals *n*_S_(0) come with broad 95% HPD intervals. The median *n*_S_(0) is between 880 and 2900 among the clusters. Cluster 1 turns out to be the most informative here, with its 95% HPD ranging from 140 to 3600 (median 880). The least informative is cluster 5 (95% HPD 180−16900, median 2900), which appears to be (*a*) sampled from the largest epidemic among the five clusters and (*b*) an epidemic for which all samples included in this analysis have been sampled before the epidemic reached its peak. Hence, one should aim to acquire samples covering as much of the duration of an epidemic as possible.

### 3.3. HCV type 2c in Argentina

**Figure 6.**
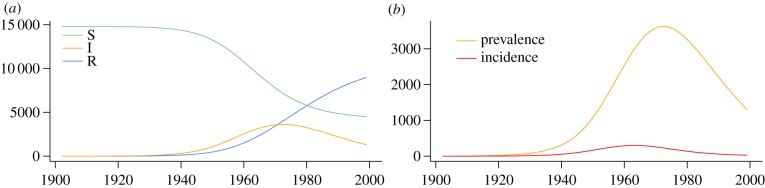
SIR trajectories and incidence of HCV-2c cluster from CdE, Córdoba, Argentina. Bayesian posterior median trajectories: the overall SIR dynamics (*a*) show that the epidemic peaked around 1970 and is declining since. Zooming into the number of infecteds, i.e. the prevalence over time in (*b*) enables comparison to the incidence. (Online version in colour.)

**Figure 7.**
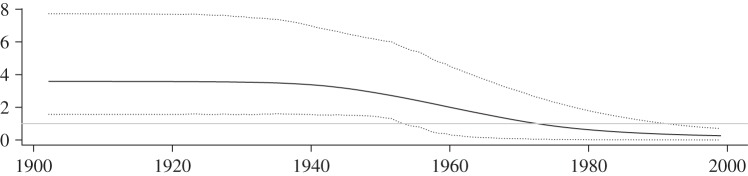
Reconstructed effective reproduction ratio—HCV-2c cluster from CdE, Córdoba, Argentina. Median effective reproduction ratio, computed from the posterior birth–death rates and SIR trajectories. Dotted lines show the 95% HPD interval.

Applied to a contemporaneously sampled HCV-2c dataset from CdE, a city in Argentina, the methods reveal that the virus caused a large local epidemic (figures 6 and 7). Despite an uninformative prior distribution on the sampling probability *ρ*, we obtain a median *ρ* = 2.6% (95% HPD: 2.3%–7.6%), which agrees very well with direct calculations based on previous estimates [20]. We estimate 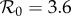 (95% HPD: 1.6–7.7), *n*_S_(0) = 14 800 (3200–29 600) and *γ* = 0.056 (95% HPD: 0.014–0.134), the latter indicating an infectious period of 17.7 years. The time of origin of the local epidemic in CdE is estimated to be 1906, with the root of the tree being placed in 1914.

For the sake of comparability, we also analysed the larger dataset (including another 29 sequences from places within Córdoba province) that was investigated by Dearlove & Wilson [21]. Initially, we employed uninformative prior distributions for the epidemiological parameters resulting in an estimate of the epidemic population size of *N* = 5200 (400–37 000) and a sampling proportion of *s* = 68% (27–100%). These results neither match the large population of Córdoba province (1.3 million) nor the small sampling proportion (2.8%) encountered by Mengarelli *et al*. [20]. This suggests a model misspecification. Given the large size of Córdoba province (165 km^2^), it appears that this dataset requires either the analysis of subsampled local epidemics (as we did for CdE) or the incorporation of population structure into the model. In fact, repeating the same analysis with a prior distribution that forces the sampling proportion to be small, we obtain results that are very similar to the estimates obtained by Dearlove & Wilson [21] under a coalescent SIR model (electronic supplementary material, figure S3). These results might explain why the analysis of the larger set resulted in unrealistically small estimates of the duration for the infectious period (average 1/*γ* = 1.47 years (coalescent SIR), 1/*γ* = 8.3 years (BDSIR)).

## 4. Discussion

Phylodynamic methods play an important role in understanding virus dynamics. Awareness of the interaction of evolutionary and ecological dynamics is essential for the development of containment strategies for virus outbreaks over short and long timescales. We have presented a model that couples evolutionary processes with the underlying stochastic host dynamics in order to obtain realistic estimates of the evolutionary as well as epidemiological history. Existing phylodynamic approaches often infer a phylogeny that is then assumed to be fixed for epidemiological inference [23,24] (see [2] for a review of further methods).

Our approach couples a birth–death tree prior with a compartmental epidemiological SIR model such that the epidemiological parameters are estimated simultaneously with the reconstruction of the phylogeny. This way the uncertainty of the tree is integrated into the inference of the epidemiological dynamics. The choice of the BDSKY model as a kernel for the prior on the phylogeny is natural: epidemiological parameters, for example, the basic reproduction ratio 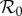, are readily computed from an appropriate parametrization and limitations of the coalescent process, for example, the deterministic population size assumption, are avoided. Note that the assumption of the BDSKY plot [8], stating that infected individuals become non-infectious upon sampling, also applies here. This is a somewhat artificial assumption made for computational convenience. To avoid such an assumption would require allowing phylogenetic trees containing ‘direct ancestors’. The first steps towards the relaxation of this assumption have recently been taken [25].

Recently, Leventhal *et al*. [26] developed a similar phylodynamic model that couples a birth–death process with a compartmental *SI* model and showed that negligence of the stochastic epidemiological dynamics can introduce bias into phylogenetic reconstruction.

Traditional coalescent-based approaches often suffer from difficulties interpreting the effective population size [27]. Explicit simulation of the stochastic SIR trajectories in the BDSIR model yields separate estimates of incidence and prevalence. This explicit separation of incidence and prevalence facilitates correct interpretation of results, although one must still take quantities, such as offspring distribution, population structure and selection pressures, into account. Nevertheless, the resulting trajectories provide information about features, for example, the time of the epidemic peak. Alternatives to the independence MH sampler used to sample the stochastic SIR trajectories, such as particle filtering [23] or pure Monte Carlo methods, might yield some computational benefit, but at the expense of the inference of the marginal posterior distribution of the compartment trajectories.

A promising coalescent-based phylodynamic model that incorporates complex population dynamics was developed by Volz [24]. However, it still assumes a deterministically changing population size. In fact, when applied in [28], it is based on a fixed phylogeny that has presumably been reconstructed based on a standard coalescent tree prior. However, note that Volz [24] could be extended to take into account stochastic epidemiological dynamics in a similar manner to that employed for the BDSIR model. If stochastic trajectories were used for the coalescent rates and implemented in a Bayesian framework it would enable direct comparison between birth–death methods and the coalescent-based methods described in [24].

In our simulation study, we have shown that the BDSIR model accurately estimates epidemiological parameters from simulated SIR trees. We have applied the model to five genetic clusters of HIV-1 type B from the UK. The data analysis revealed the epidemic stages in which the clusters were sampled. Only cluster 1 appears to be at the end of the epidemic, while the other four clusters were sampled around the time of their peak. Surprisingly, there is considerable variation in the estimates of the basic reproduction ratio 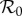 among the clusters. In cluster 3, the estimated median is 1.9, in clusters 1 and 5 it is slightly above 3. These differences in the estimated 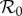 values across the five clusters, and their deviation from the common prior distribution, confirm that the sequence data contain information about the epidemiological parameters. Although we did not model variation of the underlying transmission rate among individuals, the variation of estimated epidemiological parameters among the clusters might point us towards the existence of super-spreaders.

Comparing the results of the analysis of cluster 2 to those using the BDSKY plot, published by Stadler *et al*. [8], the estimates of the sampling proportion in both analyses agree (47% here versus 50% BDSKY). Expectedly, the estimated basic reproduction ratio 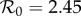 is slightly larger than the effective reproduction ratio 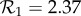 near the origin that resulted from the BDSKY analysis. Overall, analysis under the parametric BDSIR method resulted in narrower HPD intervals than that under the non-parametric BDSKY method, with the BDSIR intervals being contained in the BDSKY intervals.

The analysis of 44 HCV-2c sequences from the city of CdE supports the theory that this genotype has been introduced to Argentina during a European immigration wave between 1880 and 1920, as the most recent common ancestor of the sample analysed here is placed in this period. From the CdE subset, we have estimated an average duration of infectiousness of 17.7 years, which agrees with the 10–30 year range that has previously been supposed [29].

In conclusion, the BDSIR model provides the ability to simultaneously reconstruct evolutionary processes with their underlying host population dynamics from viral sequence data, and in particular the inferred parameters allow us to make statements about the future fate of the epidemic. Although we have used strong simplifications concerning the epidemiological dynamics of viruses like HIV (e.g. [30]), this work is the first step towards more sophisticated methods, and future work shall relax the simplifying assumptions made here. We emphasize that this general technique is applicable not only to viruses but also to any rapidly evolving organism for which the evolutionary dynamics act on the same timescale as the population processes of their hosts. Future work will aim at extensions that incorporate temporal and spatial structuring of the host and/or viral population.
